# Lifestyle Risk Factors for Type 2 Diabetes Mellitus and National Diabetes Care Systems in European Countries

**DOI:** 10.3390/nu12092806

**Published:** 2020-09-13

**Authors:** Emma Altobelli, Paolo Matteo Angeletti, Valerio F. Profeta, Reimondo Petrocelli

**Affiliations:** 1 Department of Life, Health and Environmental Sciences, University of L’Aquila, 67100 L’Aquila, Italy; paolomatteoangeletti@gmail.com; 2 Epidemiology and Biostatistics Unit, Local Health Unit, 64100 Teramo, Italy; 3 Rianimazione e TIPO Cardiochirurgica, Ospedale G. Mazzini, Local Health Unit, 64100 Teramo, Italy; 4 Department of Community Health, Local Health Unit, 64100 Teramo, Italy; valerio.profeta@aslteramo.it; 5 Public Health Unit, ASREM, 86100 Campobasso, Italy; reimondo.petrocelli@asrem.org

**Keywords:** type 2 diabetes, lifestyle risk factors, EU 28, registries, health care system

## Abstract

Background. Diabetes is increasing by 3.09% per year in males and 1.92% in females. Lifestyle risk factors are related to diabetes. The aim of this work is to highlight within EU-28 countries the distribution percentages of some lifestyle risk factors and some components of diabetes health care. Methods. A literature search was conducted to highlight the presence of diabetes registries, which are fundamental tools for disease surveillance and health planning; the presence of a national diabetes plan (NDP); the care setting; and methods used for reimbursement of drugs, devices, and coverage of any comorbidities associated with diabetes. A multiple correspondence analysis (MCA) was carried out to evaluate the possible associations between the variables considered. Results. The highest percentages of diabetes (>10%) are registered in Bulgaria, Malta, and Hungary. Concerning the prevalence of overweight, no European country shows overall percentages of less than 50%. Regarding obesity, 57% of countries show prevalence rates of 25%. The record for physical inactivity belongs to Malta, with 45% of individuals being inactive. The percentage of physical inactivity for females is higher than for males across Europe. In total, 57% of the countries have an insurance-based health system, while 12 countries have public national health systems. Further, 57% of countries have an NDP, while 42% of the EU countries have established a prevalence register for diabetes. Conclusions. Prevalence rates for type 2 DM in the range of 8–9% are noted in 50% of EU-28 countries. In total, 21 out of EU countries show a high prevalence rate for overweight, while 7% of EU-28 countries have an obesity prevalence rate of 25%. Diabetes treatment is entrusted to general practitioners in most countries. The results of this work highlight the differences between countries, but also between genders.

## 1. Introduction

Type 2 diabetes mellitus represents the paradigm of chronic diseases in which there is a close association between family and environmental factors. It is now a health problem with enormous global impact, with estimates of continuous growth [1]. An important universally recognized risk factor is high calorie intake with limited intake of fiber, which results in an increased accumulation of visceral fat, an increase in body mass index (BMI), and increase in abdominal circumference [2].

Another relevant risk factor is sedentary lifestyle. Some studies have shown that mild physical activity (light walking 30 min/day) reduces the risk of diabetes by 30%. In parallel, a sedentary lifestyle can double the risk of diabetes [3].

Epidemiological surveillance of diabetes mellitus, performed through population-based registries, is important for estimating disease burden. Population-based registries are active in many European countries to evaluate the incidence of type 1 diabetes [4,5,6], while registries that estimate the prevalence of type 2 diabetes are less frequent [7,8,9,10,11,12,13,14,15,16,17,18,19,20,21,22,23,24]. The surveillance system for diabetic disease also has another important tool for programming, improving care and resulting in effective and efficient management of the disease: The National Diabetic Plan (NDP).

The goal of the European Commission, through the JA-CHORDIS project (Joint Action for Chronic Disease), was to invite countries to develop a national plan. The WHO also confirmed its commitment in 2013, which was made to the treatment of non-communicable diseases and related risk factors [25].

In the European Union (EU 28 countries), diabetes care is provided in 19 out of 28 countries by general practitioners rather than diabetes centers. As highlighted by Gupta et al. [26], a health care system that provides and assumes the costs of services and incentives for general practitioners to control diabetic disease allows cost optimization by slowing down the progression of the disease itself.

In this context, accessibility to diabetes treatment and monitoring for associated complications (insulin, oral antidiabetic drugs, tools for daily blood glucose control; laboratory monitoring of HbA1c, retinopathy, diabetic foot; monitoring of dyslipidemia and cardiovascular risk factors) improve if an active surveillance system is present.

Moreover, it is strategic and cost-effective to invest in the early stages of the disease, promoting healthy lifestyles as much as possible. In fact, a meta-analysis by Glenchner et al. shows how changes in lifestyle in people with prediabetes significantly reduces the progression of the disease, in addition to reducing costs [27].

Another objective of the WHO 2030 Agenda for Sustainable Development is the reduction of non-communicable diseases by one-third. Obesity and physical inactivity are two very important risk factors [28]. Obesity is a recognized risk factor for diabetes [29], which is increasing in Europe. Forecasts show that the trend is increasing by 3.09% per year in males and 1.92% in females. The prevalence of obesity, therefore, will be 36.6% in men and 32.0% in women by 2030 [30].

However, primary prevention policies active in Europe for the prevention of obesity and physical inactivity [31] still appear to be different.

The aims of this work are to highlight within EU 28 countries: (i) the distribution percentage of some lifestyle risk factors; (ii) the presence of diabetes registries as fundamental tools for disease burden; (iii) description and organization of health systems and occurrence measures through use of multiple correspondence analysis.

## 2. Materials and Methods

**Figure 1:**
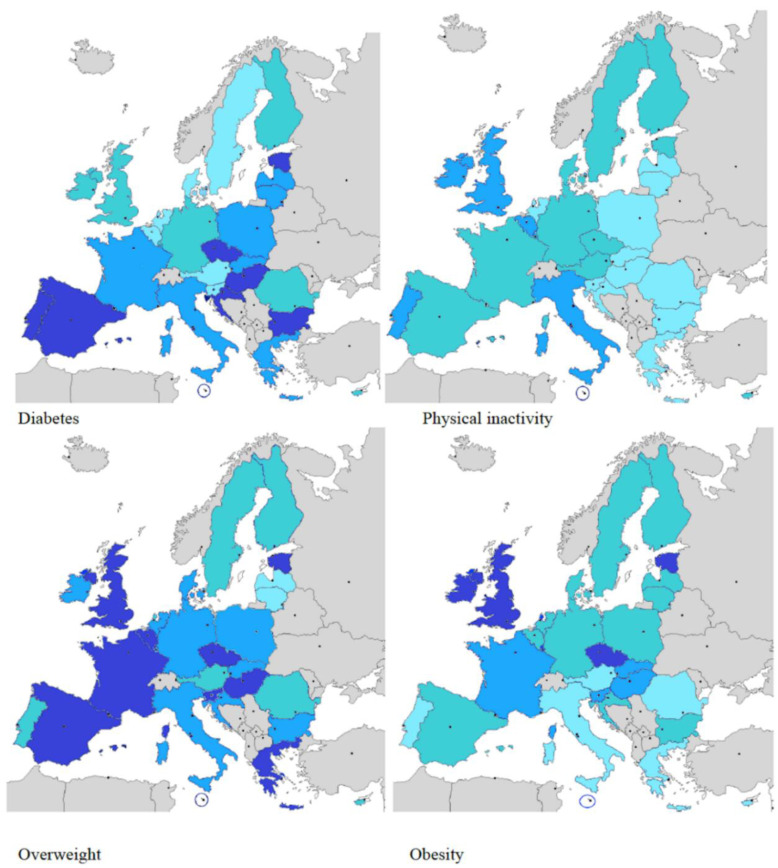
Type 2 diabetes and risk factors in males.

**Figure 2:**
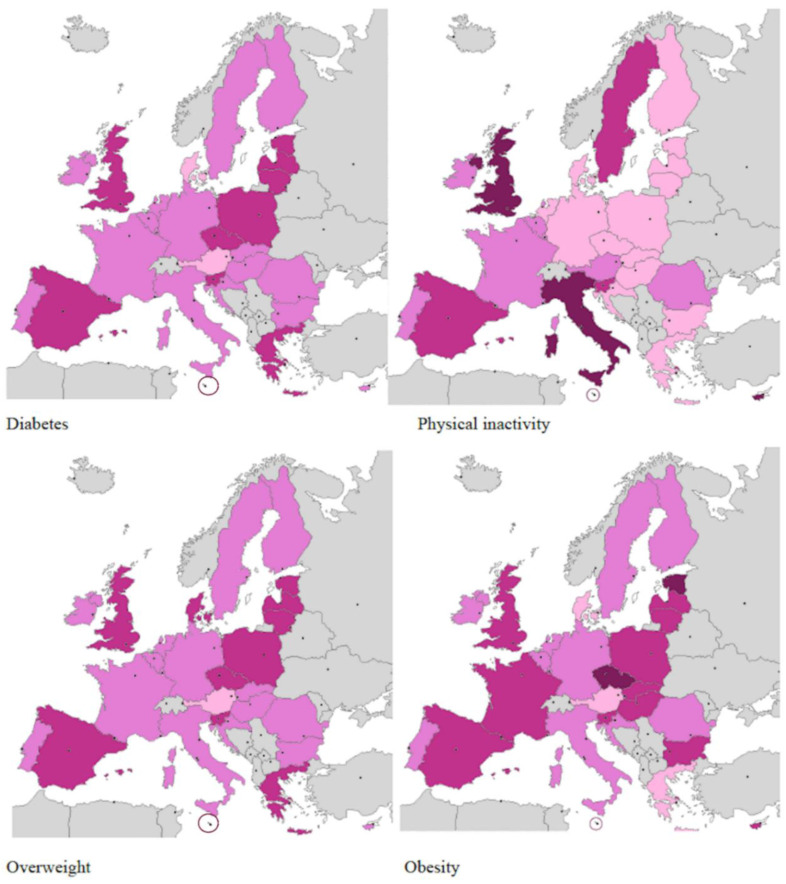
Type 2 diabetes and risk factors in females.

Table 1 and Figure 1 and Figure 2 summarize the information related to the income and distribution percentages of the following risk factors: overweight, obesity, physical inactivity, and type 2 diabetes. Data came from Eurostat datasets [32] and we used the Map Creator 2 software to build the maps (Figure 1 and Figure 2).

**Figure 3:**
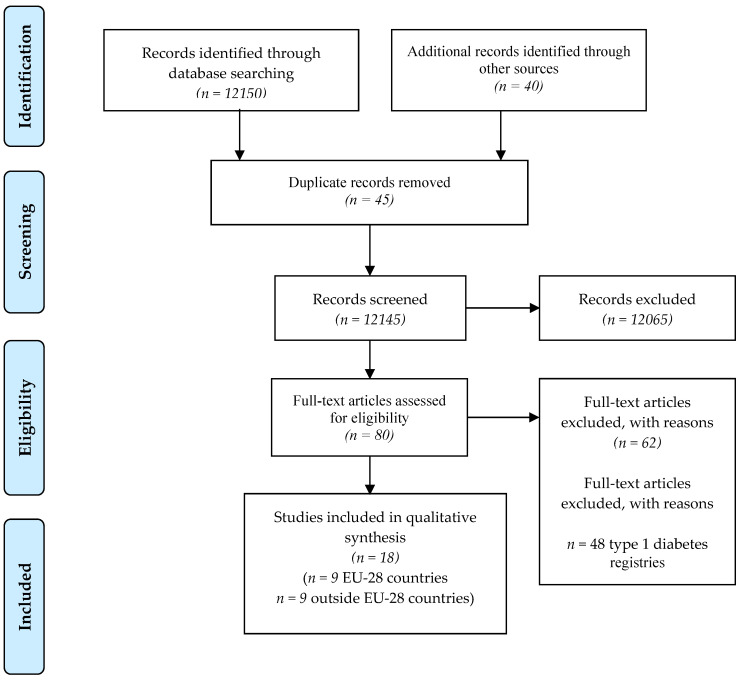
PRISMA flow chart.

A literature search was conducted to highlight the presence of diabetes registries as fundamental tools for disease surveillance and health planning (Table 2). The keywords used were registries OR incidence OR prevalence AND diabetes mellitus; insulin-dependent registries OR incidence OR population based AND diabetes mellitus, insulin-dependent; “Prevalence” OR “Registries” OR “Population Based” AND “Diabetes Mellitus, Type 2” AND “Epidemiology”; considering publications from the last 10 years, in English, over 19 years of age. The PRISMA [33] method was used to select bibliographic entries (Figure 3). Only references to type 2 diabetes registries were selected.

The data covered the type of health system, presence or absence of a national diabetes plan (NDP), presence of a population-based register, care setting, methods for reimbursement of drugs, devices and coverage of any comorbidities associated with diabetes, and the prevalence of and mortality from diabetes, gathered from the institutional sites of individual European countries to investigate the presence of national data and policies for diabetes control. Furthermore, to ensure the completeness of the data, the following sources of information were consulted: EUROSTAT [32], WHO diabetes country profiles [34], European Commission (EC) [35], International Federation of Diabetes (IFD) [1], Foundation of European Nurses for Diabetes (FEND) [36], and the World Bank [37]. All data are reported in Table 3.

A multiple correspondence analysis (MCA) was carried out in order to to evaluate the possible association between the variables taken into consideration, including EUROSTAT data for the countries of the European Union, data relating to mortality per 100,000 inhabitants and the mortality trend [32], the prevalence of diabetes [32], the organization of the health system [35,36,38], the presence of a national diabetes plan, the year of approval [35,36], the general practitioners and diabetic centers involved, and the cost percentage of diabetes of the total health expenditure [38].

The variables listed above were classified as follows: percentage of diabetes (≤6%, >6%), diabetes mortality (≤25 per 100,000, greater than >25 per 100,000), mortality trend (growth, stable and in reduction), and percentage cost of diabetes of total health expenditure (≤9%, >9%). The MCA was conducted using SAS statistical software. The graphical representation takes into account the variables that contributed most to the variance.

## 3. Results

### 3.1. Risk Factors and Diabetes

Countries belonging to the European Union show high income rates, except for Bulgaria and Romania. The highest percentages of diabetes (>10%) are registered in Bulgaria, Malta, and Hungary. Values for diabetes of between 8 and 9% are shown for 50% of the countries, including the Czech Republic, Croatia, Estonia, France, Italy, Greece, Lithuania, Latvia, Poland, Portugal, Romania, Slovakia, Slovenia, and Spain; while values between 6 and 7% are shown for Austria, Belgium, Cyprus, Denmark, Finland, Luxembourg, Germany, Ireland, Sweden, the United Kingdom, and the Netherlands. Regarding men, seven countries (25%) have a diabetes percentage rate higher than 10%: Bulgaria, Czech Republic, Estonia, Hungary, Malta, Spain. Regarding women, Bulgaria shows a rate of 10%.

Concerning overweight individuals, no European country shows overall percentages of less than 50%. In fact, as many as 21 out of 28 countries (75% of European countries) show a high percentage of overweight of 60%; the remaining 7 countries show percentages of between 50 and 60%. Overweight affects 70% of men in 6 countries (Czech Republic, Estonia, Luxembourg, Malta, Spain, and UK) and percentages between 60–70% are shown for 21 states. Only Latvia has a percentage just below 60%. The figures for women are globally similar, with the exception of Austria, where the percentage of overweight is slightly lower (49.2%). In 17 out of 28 countries, the percentage of female overweight is between 50 and 60%, while in 10 countries the percentage exceeds 60% (Czech Republic, Denmark, Estonia, Greece, Latvia, Lithuania, Malta, Poland, Spain, UK).

Regarding obesity, 57% of European countries show values of obesity of 25%: Bulgaria, Croatia, Czech Republic, Estonia, France, Greece, France, Hungary, Ireland, Latvia, Lithuania, Malta, Poland, Slovakia, Slovenia, Spain, UK). Percentages in the range of 23.4–24.5% are shown for Cyprus, Italy and Romania. The remaining countries show obesity percentages ranging between 20 and 22%. Regarding gender, obesity rates are above 28% for men in 4 countries (Czech Republic, Estonia, Luxemburg, UK), while the lowest values are recorded in Austria, Portugal, and Romania. Particularly relevant is that in some countries (Czech Republic, Malta, Estonia, and UK) the percentage of obese women is greater than 30%. Instead, the percentage exceeds 25% in 11 countries (Bulgaria, Cyprus, France, Hungary, Ireland, Lithuania, Poland, Slovakia, Slovenia, and Spain). On the other hand, the percentages of physical inactivity are more variable from country to country. The highest value for physical inactivity belongs to Malta with 45%, followed by countries in southern Europe (Italy, Spain, Portugal) and northern Europe (Belgium and the United Kingdom). It is important to underline that the percentage of physical inactivity in females is higher than in males across Europe.

### 3.2. Registries

A total of 12,150 references were identified through database searching were, while 40 were identified through manual searching. Of these, 45 references were excluded because they were duplicates. Of the remaining papers, 80 were selected as potentially valid for the systematic review. A further 14 papers were excluded for not containing the requested information. In total, 66 papers were analyzed, of which 48 covered type 1 DM and 18 covered type 2 DM (Figure 3 PRISMA Flow Chart). Of the latter 18, 9 were EU-28 countries and 9 were from outside the EU-28. All results are described in Table 2.

### 3.3. Organization of Countries and Costs

European health systems vary from country to country, and even within individual countries. However, in most European countries (57%) there are insurance-based health systems, while in 12 countries there are public national health systems. In 57% of countries, there are national diabetes plans. In total, 42% of the countries belonging to the European Union have established a prevalence register for diabetes. Diabetes care is mainly entrusted to the general practitioner in 19 countries, while in the remaining countries it is entrusted to diabetes centers. In health systems where health care is totally managed by the state government, the latter provides coverage for drugs, devices, and associated comorbidities, except for Latvia. In insurance systems, on the other hand, only in Belgium is there full coverage for expenses relating to drugs, devices, and comorbidities, while in the remaining countries there are shares for different copayments. Mortality is extremely variable; the lowest values are found in Finland, while the highest values are present in the islands Malta and Cyprus. There are only two countries with values above 40/100,000, which are Croatia and Czech Republic; while mortality values of between 30 and 40/100.00 are found in Austria, Portugal. and Hungary. Seven out of 28 countries show mortality values of between 20 and 30, including Bulgaria, Denmark, Germany, Italy, Latvia, Poland, and Sweden. In total, 42% of European countries show mortality values of between 10 and 20/100,000, including Belgium, Estonia, France, Greece, Ireland, Lithuania, Luxembourg, Romania, Slovakia, Spain, UK, and the Netherlands.

### 3.4. Multiple Correspondence Analysis

The country and year of approval of each national diabetes plan represent the first dimension, which account for about 35% of the variance; the prevalence and mortality trends represent the second dimensions, which account for around 25% of the variance.

**Figure 4:**
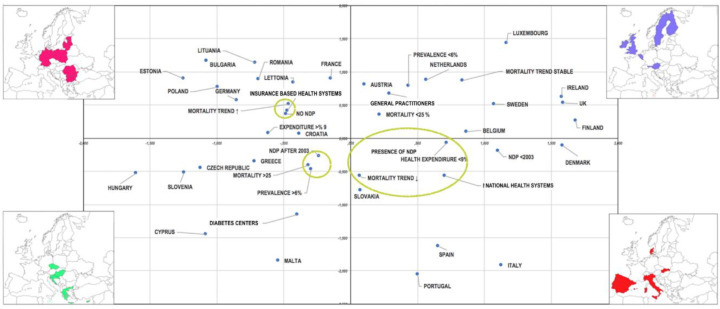
Multiple correspondence analysis. Graphical representation of the possible associations between the variables taken into consideration.

All results are represented in Figure 4.

The first quadrant includes the following countries and variables—Finland, the United Kingdom, Ireland, Sweden, Belgium, Austria, Luxembourg, the Netherlands, each showing a mortality rate below 25.0, diabetes <6%, stable mortality trend, and with diabetes care entrusted to general practitioners. In this quadrant, a cloud of points can be seen, showing mortality <25/100,000 diabetes <6% for Austria and Netherlands, with diabetes care entrusted to general practitioners.

The following countries and variables represent the second quadrant—Estonia, Bulgaria, Poland, Romania, Lithuania, Latvia, France, and Germany, each showing health expenditure for diabetes >9% compared to the total health expenditure, an increasing mortality trend, the absence of a national diabetes plan for diabetes, and health insurance. In this quadrant, an aggregation zone can be highlighted, which includes the absence of a national diabetes plan, a rising mortality trend, and health insurance.

Hungary, the Czech Republic, Slovenia, Greece, Croatia, Cyprus, Malta, and the geographical area of Eastern Europe represent the third quadrant, each having a mortality rate greater than 25%, the approval after 2003 of the NDP, a % of diabetes >6%, and the presence of diabetes services. It is important to underline that a point of clouds includes mortality greater than 25%, percentage of diabetes >6%, and approval year of the NDP after 2003.

The fourth quadrant includes Portugal, Italy, Spain, Slovak Republic, Denmark, each showing the presence of a NDP, a NDP approved before 2003, a national health system, a decreasing mortality trend, and a health expenditure for diabetes <9% of the total national health expenditure. The cloud of points represents the presence of an NDP, health expenditure for diabetes <9%, and the presence of the national health service.

## 4. Discussion

The chronicity control system is represented by the chronic care model (CCM), which was developed in the mid-1990s by Wagner [39,40]. This model covers the needs of health organizations and citizens. The CCM provides six levels of implementation: the organization of care systems with the removal of barriers; self-management with support from a caregiver; support for decisions on prevention or treatment strategies based on medical evidence; delivery of services; a system for recording and monitoring care and community and public health resources [41]. For diabetes mellitus, this means a combination of programs aimed at nutrition education, autonomous control of blood glucose with related strategies, psychological support, and personal empowerment [42].

The growth estimates for diabetes in Europe are quite clear—an increase from 59.8 million cases in 2015 to 71.1 million cases in 2040 [1].

These data are even more alarming in light of the recent SARS-Cov2 pandemic, posing a problem to healthcare stakeholders. In fact, diabetics are more susceptible to lower respiratory tract infections due to the abnormal neutrophil function induced by hyperglycemia [43]. Data from clinical studies show the increased susceptibility of diabetics affected by atypical pneumonia [44]. In this context, the enhancement of telemedicine services appears to be a priority in the control of chronic diseases, especially in lockdown periods. A recent meta-analysis has shown that telemedicine for diabetes treatment is cost effective for both retinal screening and telemonitoring [45].

Another work highlights how the control of the diabetic via telematics allows better control of glycated hemoglobin [46], which is the main marker of diabetes progression [47].

An important aspect to consider is the organization of diabetes services. The economic crisis of the period 2008–2013 severely tested the health systems of individual countries, with progressive cuts to some services or increases in copayment quotas. Some health systems, by virtue of their organization or recent reform, have been able to cope with these new economic scenarios, while others have found themselves in more difficulty; the effects of the recent economic crisis are still fully visible in terms of mortality trends [48].

The close associations between the organizational and financial aspects can be deduced from the distribution of the variables obtained from the analysis of the reports. In fact, it is clear that the activation of a National Diabetes Plan can contribute to the reduction of the prevalence of mortality from DM and the containment of the global costs of diabetes.

Our results show that in countries where there is a national health system (UK, Italy, Spain, Portugal) or an insurance system with high social protection (The Netherlands, France), excellent performance is noted in the control of diabetic disease and its comorbidities. In fact, the guarantee of access to therapies and control and prevention of complications contribute to reducing mortality, while at the same time lead to significant savings. These results are most evident in countries where a national diabetes control plan has been in place for at least 15 years.

Another aspect that emerges from our analysis is the care setting. In fact, it seems that the management of diabetes by practitioners compared to diabetic centers guarantees better results in terms of the prevalence of and mortality from T2DM, as underlined by the aforementioned meta-analysis of Gupta et al. [26]. Access to care or better delivery of care represents one of the cornerstones of the CCM model.

In our opinion, a similar system should also cover obese individuals by actively involving them in prevention policies, emphasizing self-care by self-management [49].

Our data show that obesity and being overweight are closely related to physical inactivity, especially in females, representing a gender gap. An emphasis on this theme was noted for the women’s football world championship [50]. Countries should make greater efforts to guarantee women access to sports activities, promoting the removal of sociocultural barriers and with ad hoc investments; for example, it has been shown that quality public transport and travel infrastructure for pedestrians could reduce the gender gap, allowing women to practice physical activity with greater ease and accessibility [51]. This is a fundamental aspect, especially in light of the fact that physical inactivity is one of the determinants of non-communicable diseases [52] and one of the main determinants of the increase in BMI, and therefore of obesity [50]. In fact, it has been estimated that by 2050, in EU-28 countries obesity will reduce life expectancy rates from 0.9 to 4.2 years and that 8.4% of health budgets will be used to treat complications associated with obesity. Moreover, the consequent economic effects of obesity directly reduce productive activities [51,53].

## 5. Conclusions

In conclusion, 50% of EU 28 countries show type 2 DM prevalence rates in the range of 8–9%. In addition, 21 of the EU-28 countries show a high percentage of overweight, while 7% of EU-28 countries have an obesity prevalence rate of 25%.

The record for physical inactivity belongs to Malta. In general, physical inactivity rates are higher for females than males.

Regarding care organizations, national public insurance is present in 57% of countries.

Diabetes treatment is entrusted to general practitioners in most countries.

The results of this work highlight the differences between countries, but also between genders. The patterns identified could indicate cultural and gender trends to which future public health interventions should be addressed.

Greater attention should be given to the fight against risk factors for non-communicable diseases, particularly diabetes, considering its high prevalence. This must be a priority for citizens at higher risk.

## Figures and Tables

**Table:** 

LEGEND				
Diabetes (%)	7.1–7.9	8.0–8.9	9.0–10.9	>11
Physical Inactivity (%)	<20	20.1–29.9	30–39.9	>4
Overweight (%)	<60	60.1–64.9	65–69.9	>70
Obesity (%)	<22	22.1–25.9	26–28.9	>29

**Table:** 

LEGEND				
Diabetes (%)	<6	6.1–7.9	8.0–9.9	>10
Physical Inactivity (%)	<30	30.1–34.9	35–39.9	>40
Overweight (%)	<50	50.1–59.9	60–66.9	>70
Obesity (%)	<20	20.1–24.9	25–29.9	>30

**Table 1:** Distribution percentages of risk factors considered.

Nation	Income	Overweight (%)			Obesity (%)			Physical Inactivity (%)			Diabetes (%)		
		Total	Men	Women	Total	Men	Women	Total	Men	Women	Total	Men	Women
Austria	High	56.6	64.4	49.2	20.1	22.1	18.1	26.4	21.1	31.3	6	7.1	5
Belgium	High	60.5	69	52.3	22.1	24	20.2	37.4	31.5	42.9	6.4	7.5	5.4
Bulgaria	Upper-Middle	63.6	67.8	59.7	25.6	23.6	27.5	23	19	26.7	10.3	10.7	10
Croatia	High	62.9	67.8	58.4	25.6	24.3	26.8	19.8	16.8	22.5	9.9	10.7	9.2
Cyprus	High	61.8	64.7	58.7	24.5	22.3	26.8	35.3	29.3	41.5	7.8	8.9	6.7
Czech Republic	High	67.2	72.8	61.8	29.1	28.1	30.1	26.2	24.1	28.2	9.9	10.7	9.2
Denmark	High	58.7	67.5	60	210	23.3	18.8	26.6	24.3	28.9	6.1	7.2	5
Estonia	High	67.2	72.8	61.8	29.1	28.1	30.1	26.2	24	28.2	9.6	10.2	9.1
Finland	High	59.4	65.6	53.4	22.8	23.4	22.2	26.2	24.2	28.1	7.7	8.7	6.8
France	High	64.1	69.6	58.6	25.7	25.3	26.1	26.4	21.2	31.2	8	9.5	6.6
Germany	High	59.7	67	52.7	22.7	24.1	21.4	23.4	20.1	26.5	7.4	8.4	6.4
Greece	High	64.29	69.6	60.2	25.1	23.6	18.2	15.4	12.4	15.4	9.1	9.5	8.8
Hungary	High	63.3	69.4	57.8	26	25.5	26.5	20	18.5	23	10	10.6	9.4
Ireland	High	62.9	68.8	57.1	27	27.3	26.8	36	30.6	41.2	7.3	8.4	6.2
Italy	High	64	68.7	59.5	23.7	22.5	24.8	35.9	30	41.3	8.5	9.6	7.4
Latvia	High	64.3	59.1	61.5	25.6	23.2	27.7	23.8	19.3	27.3	9.4	9.2	9.6
Lithuania	High	62.8	64.2	61.7	27.5	23.2	27.7	23.8	19.3	27.3	9.7	9.8	9.5
Luxemburg	High	61	70.7	51.5	24.8	28.3	21.3	30	28.2	31.7	6.8	8.3	5.3
Malta	High	69.6	71.6	63.7	28.7	26.2	31.1	45.2	40.3	49.4	10.1	11.4	8.8
Poland	High	64.2	68.2	60.5	27	24.8	29.1	20.5	14.4	26	9.5	9.8	9.3
Portugal	High	59.8	65	55	22.1	21.4	22.8	37.3	33.5	40.8	9.2	10.7	7.8
Romania	Upper-Middle	60.8	65.2	56.5	23.4	21.8	24.9	26.5	19.1	33.3	8.4	8.5	8.4
Slovakia	High	64	68.5	59.7	27.4	25.9	28.3	19.2	16.8	21.4	9.5	9.8	9.8
Slovenia	High	64.8	69.9	59.7	27.4	26.7	28.2	24.1	18.5	29.2	8.6	9.2	8.1
Spain	High	65.6	70.3	60.9	26.5	24.9	28	33.4	29.2	37.4	9.4	10.6	8.2
Sweden	High	59.2	65.9	52.4	22	23.6	20.4	31.1	26.2	35.8	6.9	7.8	6
The Netherlands	High	59.8	67.2	52.6	21.9	23.2	20.6	19.9	16	19.7	6.1	7.0	6.1
United Kingdom	High	66.7	71.1	62.4	29.8	28.5	31.1	40.4	35.4	44.3	7.7	5.4	6.9

**Table 2:** Type 2 diabetes registries.

Country	% Case Ascertainment	Year	Primary Source	Secondary Source	Others Sources	Prevalence		
						Male	Female	Total
**EU-28**								
Denmark [7]	100%	2007	Hospital database	Pharmaceutical database		4.3	4.1	4.2
Spain, Catalonia [8]	-	2009	SIDIAP database (general practitioners)	Pharmaceutical database		-	-	7.6
Italy, Reggio-Emilia [9]	80%	2009	Medical tax exemption database	Hospital database	Hospital database	5.3	4.4	4.8
Germany [10]		2010	Medical database	Pharmaceutical database		7.4	6.9	-
Portugal [11]	-	2012	-	-	-	-	-	12.7
Finland, North Karelia [12]	-	2012	Regional surveillance system	Laboratory database		-	-	6.2
Italy, Piedmont [13]	79%	2013	Regional surveillance system	Medical tax exemption database	Hospital database	4.6	4.8	-
Italy, ARNO Consortium [14]	-	2015	Pharmaceutical database	Hospital database	Medical tax exemption database	-	-	6.2
Sweden [15]		2015	Pharmaceutical database	Hospital database		7.9	5.8	6.8
**OUTSIDE EU-28**								
Hong Kong [16]	-	1999–2008	Insurance database	Laboratory database	pharmacological prescriptions	11.4	9.3	10.4
Canada [17]	-	2009	National surveillance diabetes system	Insurance database	Hospital database	-	-	6.4
Singapore [18]	-	2010	Insurance database	Laboratory database on HbA1c, pharmacological prescriptions	Insurance database	-	-	13.3
Malesia [19]	-	2011	Patients registration to surveillance system	Medical follow-up	Clinical audit, laboratory database on HbA1c, pharmacological prescriptions	-	-	15.2
Israel [20]	-	2014	Laboratory database on HbA1c	Pharmacological prescriptions		-	-	9.0
Norway [21]	-	2014	Database Noklus (general practitioners and diabetologist)	Laboratory database	Pharmaceutical database	6.8	5.3	6.1
United States [22]	-	2015	National surveillance diabetes system			9.4	9.2	9.3
Australia [23]	-	2016	National surveillance diabetes system	Country or regional database	National survey	6.8	5.4	6.1
Russian Federation [24]	-	2016	National surveillance diabetes system	National statistical data		4.7	6.1	5.5

**LEGEND**. Data not reported: -.

**Table 3:** Description and organization of health systems and occurrence measures.

Nation	Health System	National Diabetic Plan (NDP)	Registry	Setting	Drugs Reimbursement	Device Reimbursement	Coverage of Comorbidity	Prevalence/1000	Mortality/100.000
Austria	Health Insurance System	Yes	No	General Practitioners	Total	Total	Partial	4.9	39.0
Belgium	Health Insurance System	Yes	No	General Practitioners	Total	Total	Total	6.3	12.9
Bulgaria	Health Insurance System	No	No	General Practitioners	Partial	Partial	Partial	5.3	22.6
Croatia	National Health System	Yes	Yes	General Practitioners	Total	Total	Total	7.1	47.7
Cyprus	National Health System	No	Yes	Diabetes Centers	Partial	Partial	Partial	6.1	50.3
Czech Republic	Health Insurance System	Yes	No	General Practitioners	Partial	Partial	Partial	7.7	43.7
Denmark	National Health System	Yes	Yes	General Practitioners	Total	Total	Total	4.6	27.3
Estonia	Health Insurance System	No	No	General Practitioners	Partial	Partial	Partial	7.2	14.2
Finland	National Health System	Yes	Yes	General Practitioners	Total	Total	Total	7.7	9.7
France	Health Insurance System	No	No	General Practitioners	Partial	Partial	Partial	10.0	16.7
Germany	Health Insurance System	No	Yes	General Practitioners	Partial	Partial	Partial	7.2	25.4
Greece	National Health System	Yes	No	Diabetes Centers	Partial	Partial	Partial	9.2	14.1
Hungary	Health Insurance System	No	No	Diabetes Centers	Partial	Partial	Partial	8.1	30.6
Ireland	National Health System	Yes	No	General Practitioners	Total	Total	Total	5.5	17.5
Italy	National Health System	Yes	yes	Diabetes Centers	Total	Total	Total	6.7	28.8
Latvia	National Health System	No	Yes	General Practitioners	Partial	Partial	Partial	4.7	28.0
Lithuania	Health Insurance System	Yes	No	General Practitioners	Partial	Partial	Partial	4.4	10.6
Luxemburg	Health Insurance System	No	No	General Practitioners	Total	Total	Total	5.6	14.0
Malta	Health Insurance System	Yes	No	Diabetes Centers	Partial	Total	Partial	8.3	44.6
Poland	Health Insurance System	Yes	No	General Practitioners	Partial	Partial	Partial	6.6	26.1
Romania	Health Insurance System	Yes	Yes	General Practitioners	Partial	Partial	Partial	5.3	14.4
Slovakia	Health Insurance System	Yes	No	Diabetes Centers	Total	Total	Partial	6.9	18.7
Slovenia	Health Insurance System	Yes	No	Diabetes Centers	Partial	Partial	Partial	6.9	15.21
Portugal	National Health System	Yes	Yes	Diabetes Centers	Total	Total	Total	9.3	38.7
Spain	National Health System	Yes	Yes	Diabetes Centers	Total	Total	Total	6.8	18.6
Sweden	National Health System	No	Yes	General Practitioners	Total	Total	Total	4.8	20.8
The Netherlands	Health Insurance System	Yes	No	General Practitioners	Total	Partial	Partial	5.3	19.2
United Kingdom	National Health System	Yes	Yes	General Practitioners	Total	Total	Total	5.8	11.2
